# A seroprevalence study indicates a high proportion of clinically undiagnosed MPXV infections in men who have sex with men in Berlin, Germany

**DOI:** 10.1186/s12879-024-10066-z

**Published:** 2024-10-14

**Authors:** Ulrich Marcus, Janine Michel, Nikolay Lunchenkov, Denis Beslic, Fridolin Treindl, Rebecca Surtees, Christoph Weber, Axel Baumgarten, Andreas Nitsche, Daniel Stern

**Affiliations:** 1 Department of Infectious Disease Epidemiology, Robert Koch Institute, Berlin, Germany; 2 Centre for Biological Threats and Special Pathogens German Consultant Laboratory for Poxviruses Highly Pathogenic Viruses (ZBS 1) WHO Collaboration Center for Emerging Threats and Special Pathogens, Berlin, Germany; 3 Technical University of Munich, TUM School of Social Sciences and Technology, Munich, Germany; 4 Centre for Biological Threats and Special Pathogens, Biological Toxins (ZBS 3), Robert Koch Institute, Berlin, Germany; 5 Centre for Artificial Intelligence in Public Health Research, ZKI-PH 3, Wildau, Germany; 6 Checkpoint BLN, Berlin, Germany; 7 MVZ zibp, Berlin, Germany

**Keywords:** Mpox, seroprevalence study, men having sex with men, MVA vaccination, orthopoxvirus antibodies, ATI-N antibodies

## Abstract

**Introduction:**

During the mpox outbreak in 2022, the highest number of cases in Germany were registered in Berlin, almost all of them in men who have sex with men (MSM). However, the frequency of clinically undiagnosed infections is unknown.

**Methods:**

A cross-sectional study was conducted among MSM in Berlin, Germany. Participants were recruited from private practices and community-based checkpoints specialised in HIV and STI care for MSM. They were asked to complete an online questionnaire on socio-demographic data, mpox diagnosis, vaccination history and sexual behaviour, and to provide a blood sample for serological analysis. The samples were tested for antibodies against a range of antigens to distinguish between antibodies induced by mpox infection and MVA vaccination, with pre-immune sera from childhood smallpox vaccination as a confounding factor. Associations of behavioural variables with reported and suspected mpox diagnosis as the outcome were tested using univariable and multivariable logistic regression models.

**Results:**

Between the 11th April and 1st July 2023, 1,119 participants were recruited in eight private practices and two community-based checkpoints in Berlin. All participants provided a blood sample for serological testing. Information for the online questionnaire was provided by 728 participants; core data on age and mpox history for participants who did not provide questionnaire data were provided by the practices for an additional 218 participants. A previous diagnosis of mpox was reported for/by 70 participants (7.4%). Using a conservative and strict case definition, we serologically identified an additional 91 individuals with suspected undiagnosed mpox infection. Individuals with reported or suspected mpox infections reported more condomless anal sex partners in the past 3 months (OR = 5.93; 95% CI 2.10-18.35 for 5–10 partners; OR = 9.53; 95% CI 2.72–37.54 for > 10 partners) and were more likely to report sexual contact with partners diagnosed with mpox (OR = 2.87; 95% CI 1.39–5.84).

**Conclusion:**

A substantial proportion of mpox infections were clinically undiagnosed. The number of condomless anal sex partners was strongly associated with both confirmed and suspected undiagnosed mpox infection. Therefore, mpox control measures based on clinical diagnosis of mpox are likely to have limited effectiveness in preventing mpox transmission in outbreak situations because many infections remain unrecognised and undiagnosed.

**Supplementary Information:**

The online version contains supplementary material available at 10.1186/s12879-024-10066-z.

## Background

Mpox (formerly known as monkeypox) is caused by the monkeypox virus (MPXV), a virus of the genus *Orthopoxvirus* (OPXV) within the family *Poxviridae*. Two genetically distinguishable clades have been known, clade I circulating in Central Africa and clade II present in Western Africa. Earlier epidemiological data and animal experiments suggest a lower pathogenicity of clade II mpox viruses in humans [1].

No cases were reported outside Africa until 2003, when a cluster of 47 laboratory confirmed cases associated with infected pet prairie dogs was described in the United States [2]. Further outbreaks with usually individual cases were subsequently reported in the United Kingdom, Israel and Singapore [3]. In May 2022, an mpox outbreak was recognized in Europe which has subsequently spread to more than 100 countries and territories from all six World Health Organisation regions [4] and was caused by a clade II related virus referred to as clade IIb. There are some striking features that make this outbreak rather unusual compared with previous outbreaks, including a shift in the average age and most affected age group, the sex/gender affected, risk factors, clinical course, presentation, and mode of transmission [5–7].

**Fig. 1:**
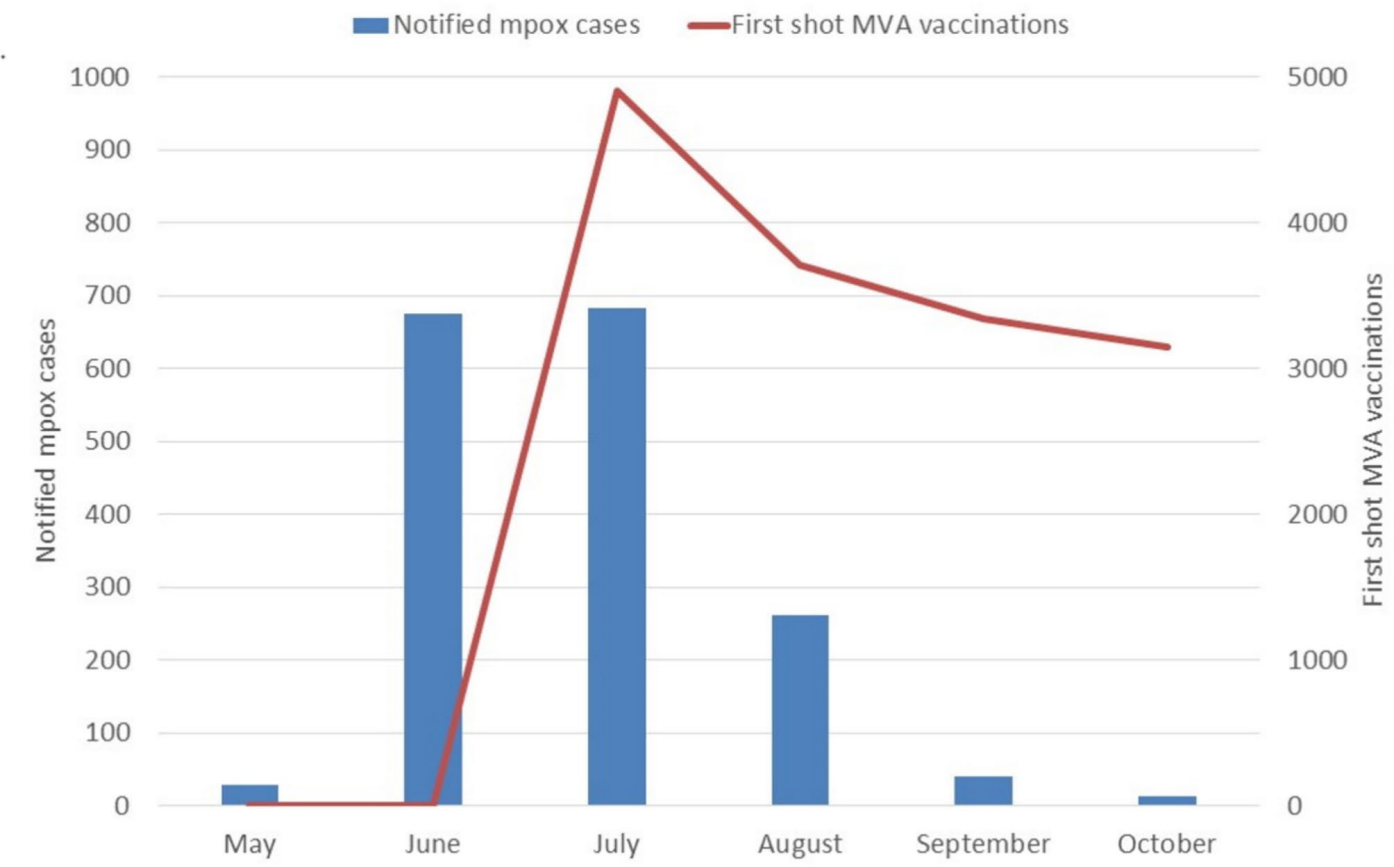
Notified mpox cases and first shot MVA vaccinations in Berlin, May–October, 2022

The first cases of mpox in Germany were identified in May 2022 [8]. To date, approximately 3,800 cases have been reported to the German mandatory surveillance system at the Robert Koch Institute (RKI), the majority of which (approximately 3,600 cases) occurred from early summer to autumn 2022. Berlin is the most affected city, with a cumulative total of 1,762 cases by the 15th April 2024. After an initial sharp increase, the number of cases decreased significantly from August 2022 onwards. Only a few cases were reported in October 2022, and no cases were recorded between January 2023 and July 2023. Since August 2023, mpox cases have been reported again in several federal states (especially in Berlin), but the numbers are significantly lower than in 2022. No deaths associated with mpox infection have been reported in Germany. As part of the emergency response, mass vaccination with Modified Vaccinia Virus Ankara (MVA)-based vaccines began in July 2022 [9, 10]. These vaccines, marketed as Imvanex (EU), Jynneos (USA) or Imvamune (Canada), are third-generation vaccines using non-replicating live attenuated viruses. They have been shown to induce a robust and protective immune response against mpox [11] and have an improved safety profile compared to second-generation replicating vaccines, which in rare cases have caused serious adverse reactions. Figure 1 shows the number of reported mpox cases and first MVA-based vaccinations in Berlin from May to October 2022 [12]. By end of October 2022 slightly more than 15,000 first shot vaccinations and 4,300 second shot vaccinations had been administered in Berlin. The number of self-defined gay men living in Berlin has been estimated at approximately 60,000, the number of gay men at increased risk of MPXV acquisition (based on a number of more than 5 sexual partners in the previous 12 months) has been estimated at 22,000 [13].

The epidemiology and clinical presentation of mpox in the current outbreak clearly suggest that the virus behaves like a sexually transmitted infection. Throughout the outbreak, sexual venues and festivals have been associated with chains of transmission [14, 15]. Primary lesions were often localised in the anogenital region (73–94%) or oropharynx, with other manifestations such as lymphadenopathy (56–85%) and fever (53–72%) often occurring after the onset of cutaneous lesions [15, 16]. Painful proctitis has been reported as a serious complication leading to hospitalisation [15]. Concomitant sexually transmitted infections were frequently observed (15–29%). These observations, together with data on high viral loads detected in genital lesions, support skin-to-skin/mucosa-to-mucosa contact during sexual intercourse as a common route of MPXV transmission. Whether genital secretions (e.g. semen) led to sexual transmission before lesions appeared or after healing, and the duration of cutaneous/mucosal shedding of infectious virus, remain unknown. However, replication competent virus can be isolated from semen as well as urine.

Several groups have described asymptomatic or clinically inapparent MPXV infections in male sexual health clinic attendees screened for gonorrhoea and chlamydia, or at baseline serological screening in MSM presenting for MVA vaccination. In these asymptomatic cases MPXV DNA has been identified in mucosal swabs taken for screening using orthopox virus-specific PCR assays [17–22]. There is a high degree of homology between antigens from different orthopoxvirus species [23–25]. This makes it difficult to distinguish the cross-reactive antibody immune response after MVA vaccination from an MPXV infection. In addition, the immune response to multiple viral proteins is complex and long-lasting. For example, antibodies from childhood smallpox vaccination can be detected decades after vaccination [26], but the extent to which they provide protection against MPXV infection is unclear [23–32]. The antibody response to vaccination with attenuated MVA is weaker compared to infection and is dependent on the number of booster vaccinations and previous childhood smallpox vaccination, declining to a relatively stable baseline level within 3–6 months, but cellular immune mechanisms appear to provide good protection, although the duration of protection is unknown [33, 34].

In differentiating between vaccine-induced and infection-induced antibodies, several scenarios need to be considered.

First, orthopoxvirus-specific antibodies may be derived from childhood smallpox vaccination and may be detectable at low levels in older individuals even decades after vaccination. The validity of self-reported childhood smallpox vaccination may be low, making it difficult to classify individuals as vaccinated. However, as childhood vaccination was mandatory worldwide until 1980, with some variation in the exact date of discontinuation, age may serve as a reliable proxy for classifying individuals as likely vaccinated or likely naïve.

Second, orthopoxvirus-specific antibodies can be induced by MVA vaccination. In contrast to self-reported smallpox vaccination, the validity of self-reported MVA vaccination may be high, especially as MVA was only made available during the 2022 outbreak and there was a widespread shortage of vaccine at the beginning of the outbreak. In addition, MVA vaccination of an individual who has been vaccinated against smallpox is thought to lead to a boost in immune responses, including both cellular and humoral immune responses [35, 36]. In contrast, individuals vaccinated with MVA alone may require at least two doses of vaccine to achieve longer lasting antibody responses [31, 33].

Finally, individuals with a clinically diagnosed MPXV infection (usually confirmed by detection of MPXV DNA in samples taken from skin or mucocutaneous lesions) are expected to mount a robust cellular and humoral immune response, but the duration and pattern of the antibody response is still under investigation [11].

Previously, we developed a robust machine learning (ML)-guided serological assay to discriminate between MVA immunised individuals, those infected with MPXV and those with no pre-existing immunity, considering childhood vaccination as a confounding factor in all groups. The assay measures immune responses to 15 OPXV-specific antigens, with 13 antigens used to train and test different ML algorithms [37].

In that study, we confirmed that the majority of individuals with undiagnosed MPXV infection are likely to mount an immune response against the ATI-N antigen [31], which can be expressed only in replicating OPXV and not in the replication-deficient MVA strains used for vaccination due to its loss during attenuation. Therefore, individuals vaccinated with MVA are not expected to react to this antigen unless they have received a childhood smallpox vaccination. This finding is crucial for the detection of undiagnosed MPXV infections in people who have received the MVA vaccination. In addition, we confirmed that the MPXV E8L antigen contributes significantly to detection of orthopoxvirus contact by infection or immunization. Both ATI-N and E8L have previously been described as excellent antigens for detecting and/or differentiating between naïve individuals and those immunised with Dryvax^®^ or MVA [31]. Therefore, that assay can be used to serologically discriminate between MVA-immunised and MPXV-infected individuals, making it highly useful for determining unrecognised MPXV infections in a population also vaccinated with MVA, as was the case in the MSM population in Berlin after the 2022 outbreak.

Our main research question was: How many clinically undiagnosed MPXV infections occurred among MSM in Berlin during the 2022 mpox outbreak?

## Methods

### Population and samples for analysis

Men and trans individuals who have sex with men were recruited in private practices and community-based checkpoints specialised in HIV and STI care in Berlin from April to June 2023. Participants were asked to complete an online questionnaire on socio-demographic data, mpox diagnosis, vaccination history, and sexual behaviour, and to provide a blood sample for serological analysis. The samples were tested for antibodies against a range of antigens to distinguish between antibodies induced by MPXV infection, MVA vaccination and childhood smallpox vaccination. An English version of the online questionnaire is available as Supplementary File 1.

We stratified the sample by age group into a subgroup younger than 50 years and a subgroup 50 years and older, to account for confounding antibodies from the childhood smallpox vaccination, which was discontinued in West Germany from 1976 on and in East Germany in 1982. This may cause misclassification of some individuals in the age group 40–49, especially if they grew up in East Germany or other Eastern European countries that still had childhood vaccination. However, the majority of individuals will be correctly classified as having received a smallpox vaccination at this age (born 1974 or earlier: presumably vaccinated).

### Laboratory testing

Serum samples were tested against a panel of 15 OPXV-specific antigens using a bead-based multiplex assay to detect OPXV-specific antibody immune responses (IgG, IgM) [37]. Briefly, 15 OPXV-specific antigens, including 5 pairs of vaccinia virus and MPXV homologous proteins, were coupled to MagPlex^®^ beads and included in a 19-plex assay with all necessary controls (positive, negative, IgG and IgM isotype controls). Two serum dilutions (1:100 and 1:1000) were used to quantify IgG and IgM antibodies, with vaccinia immune globulin (VIG; BEI Resources) used as the standard for quantification.

Population-level data were used to establish cut-off values for the E8L and ATI-N proteins to determine the OPXV-specific serostatus (positive or negative), allowing for potential misclassification due to asymptomatic infection in younger unvaccinated or MVA-vaccinated individuals. For this purpose, a serum panel of patients younger than 40 years of age was used. Naïve patients with samples collected before the mpox outbreak and without known OPXV infections were considered seronegative (*n* = 44), while MPXV-infected patients from the study were considered seropositive for the determination of the E8L-specific cut-off (*n* = 41). For the ATI-N-specific cut-off, sera from MVA-vaccinated individuals from a controlled study population prior to the mpox outbreak were considered seronegative (*n* = 13) in conjunction with the naïve sera used to determine the E8L cut-off (*n* = 44, same panel as above), while again MPXV-infected patients from the study were considered seropositive (*n* = 41, same panel as above).

### Case definition

We used the following case definitions for confirmed/ serologically suspected MPXV infection:

We defined a case as confirmed if a diagnosis of mpox was self-reported in the questionnaire or reported by the recruitment site.

We defined a case as serologically suspected.

1. If no mpox diagnosis and no MVA vaccination were reported (neither in the questionnaire nor by the recruitment centre).
  - If the age was less than 50 years; and.
  - If antibodies to the OPXV E8 surface protein were detected.
2. If vaccinated against MVA.
  - If, in addition to antibodies against the E8 surface protein, antibodies to the ATI-N OPXV protein were detected at a detection threshold that had a specificity of 95% and a sensitivity of 41% as determined by receiver operating characteristic analysis for the population-based cut-off.

This means that our case definition likely underestimates the number of mpox infections in participants who had received MVA vaccination(s).

This case definition was used to determine the study subsample that was used to analyse the behavioural correlates of an mpox diagnosis.

To approximate the maximum number of possible mpox infections in our study population we also defined an upper bound of possible mpox infections in MVA vaccinees, individuals aged 50 years and older who were likely to have received childhood smallpox vaccination, and participants of unknown age and missing questionnaire data. This upper bound was defined:

- in MVA recipients as serological detection of E8 antibodies, and ATI-N antibodies above a threshold with 77% sensitivity and 78% specificity;
- in individuals aged 50 years or older with no clinical diagnosis of mpox, as serological detection of ATI-N antibodies above a threshold with 100% specificity;
- in individuals of unknown age and without questionnaire data by serologically detecting E8 and ATI-N antibodies above a threshold with 77% sensitivity and 78% specificity.

A supplemental Table summarizing the case definition, the upper bound definition, and the expected serological profile in older age groups with smallpox childhood vaccination is available as Supplementary Table S1.

### Analysis of OPXV antibody responses by subgroups and stratified by age

We evaluated serological results for three subgroups of seroprevalence study participants, based on reported exposure, and stratified by age group. The three subgroups were (1) participants with self- and/or physician-reported mpox diagnosis (mpox reported); (2) Participants who reported MVA vaccination (MVA); and (3) Participants who denied mpox diagnosis and MVA vaccination (presumed naïve). The antibody response to the OPXV antigen ATI-N, which is expressed in MPXV but not in MVA, was used to further classify immune responses in MVA vaccinees and study participants 50 years of age and above.

### Statistical analysis

Associations between demographic and behavioural characteristics and a binary outcome variable of reported/suspected mpox infection based on our case definition were tested in a multivariable logistic regression model. The analysed group was restricted to participants with questionnaire data and under 50 years of age. MVA recipients who did not meet the 95% specificity threshold but were above the 77% specificity threshold for ATI-N antibody detection were also excluded from the analysis to reduce the risk of biasing the results due to inclusion of falsely positive and falsely negative classified participants. In this group we tested associations between reported/suspected mpox infection and reported number of partners, number of condomless anal sex partners in the past 3 months, location of sexual encounters, social and sexual contact with known mpox-infected partners, HIV co-infection and PrEP use, in univariable and multivariable regression analysis. All statistical analyses were conducted in R (version 4.3.0).

### Ethics approval

The Ethics Committee of the Berlin Medical Association approved the seroprevalence study on 14 February 2023 (Eth-40/22). All study participants gave their informed consent. Participants did not receive any incentives.

## Results

We collected 1,119 blood samples from study participants and tested them for anti-OPXV antibodies using a multiplex assay containing various OPXV-specific antigens.

### Detection of anti-orthopoxvirus antibodies

Anti-OPXV antibodies (antibodies against the E8 surface antigen) were detected in the serum of 775 of the 1119 study participants (69%). Table 1 shows the distribution of detectable antibodies in participants with a reported mpox diagnosis, reported MVA vaccination, and in participants who reported neither infection nor vaccination. The latter group was stratified by age (under 50 vs. 50 years and older) to account for possible antibodies induced by smallpox vaccination in childhood.

Data on mpox diagnosis, MVA vaccination and childhood smallpox vaccination were collected from 728 study participants using an online questionnaire. Data on clinically documented mpox diagnosis and age from study participants without online questionnaires were provided by recruiting practices for a further 218 participants, and data on clinically documented MVA vaccination for 117 study participants.

**Table 1:** Prevalence of anti-OPXV (E8) antibodies (ab) stratified by potential antibody source

	anti-E8 seropositive/group total (percent)
Total	**775/1**,**119 (69%)**
Reported mpox infection	69/70 (99%)
Reported MVA vaccination	449/507 (89%)
vaccinated once with MVA	89/124 (71%)
vaccinated twice with MVA	355/376 (94%)
No data	5/7 (71%)
Neither infection nor vaccinated with MVA	138/366 (38%)
Older than 50 years	72/81 (89%)
50 or younger	66/285 (23%)
No data	119/176 (68%)

Anti-OPXV E8 ab were detected in 69/70 (99%) study participants with reported mpox infection, in 314/334 (94%) study participants younger than 50 years with two reported MVA vaccinations, and in 62/97 (64%) of those with one MVA vaccination.

The detection of anti-OPXV ab in study participants without a history of mpox diagnosis or MVA vaccination, and younger than 50 years suggests possible undiagnosed mpox infections in this group.

To investigate this further, the antibody response to the OPXV antigen ATI-N, which is expressed in MPXV but not in MVA, was used to further classify immune responses.

Table 2 shows a cross-tabulation of (self-)reported data on mpox diagnosis and MVA vaccination history with serological predictions based on our case definition.

**Table 2:** Self-reported status, serological prediction and preliminary estimate of history of mpox infection

		mpox estimated upper bound (all age groups)	Serological prediction in study participants < 50 years			Serological prediction in study participants ≥ 50 years (*n* = 173) (possible smallpox childhood vaccination antibodies)		
Classification	Self-reported	mpox	mpox*	MVA	Naïve	mpox	MVA/smallpox	Naïve
mpox	**70**	**70**	**61**	**0**	**1**	**8**	**0**	**0**
MVA	**507**	90**	22*	346	55	10**	71	3
Presumed naïve	**366**	78**	66*	0	219	12**	60	9
No data	**176**	38**		81	57			
Total	**1.119**	276**	149*	427	332	30**	131	12

* Mpox prediction based on seropositivity for E8 (subgroup Presumed naïve) or E8 + ATI-N (subgroup MVA) antibodies according to case definition **upper bound of possible mpox infections in these subgroups

### Mpox

The results for self-reported mpox diagnoses show that serological testing was able to identify almost all (99%) reported clinically diagnosed mpox infections on the basis of E8 seroreactivity. Only one clinically diagnosed mpox infection had antibodies to E8 below the positivity threshold.

### MVA

The largest group in our study sample were individuals without a clinical diagnosis of mpox who reported MVA vaccination (one or two shots, with or without a history of childhood smallpox vaccination). In individuals younger than 50 years, our antibody assay identified 80 (19%) with antibodies to ATI-N (= upper bound). However, because of the performance of the assay, with some expected false positives, we used a stricter case definition for this subgroup with a specificity of 95%, but also a lower sensitivity (41%), leaving 22 cases meeting this stricter case definition for serological diagnosis of mpox infection before or after MVA vaccination.

Of the eight cases for which we have information that they had been diagnosed with mpox and had received the MVA vaccination, four met the strict case definition for MVA recipients with 95% specificity for ATI-N, and antibodies against ATI-N (= upper bound) were detected in seven.

### Presumed naïve

Sixty-six (23%) participants aged less than 50 years were classified as having a suspected mpox infection based on anti-E8 antibodies. Self-reported immunologically naïve participants who immunologically recognise E8 and have not received a childhood smallpox vaccination must have been exposed to an OPXV, most likely MPXV, as the performance of the assay was 100% sensitivity and 100% specificity based on the population-based cut-off values.

### Age group 50 years and older

Among individuals aged 50 years and older who reported having been vaccinated with MVA, we found serological reactivity to E8 in 81 of 84 individuals (96%), which was higher than in the younger age group (87%). The higher prevalence in the older group is probably an indication of increased reactivity due to childhood smallpox vaccination. Using the highest possible specificity threshold of 100% for ATI-N reactivity as a surrogate for differentiating between mpox infection and smallpox vaccination, 10 of 84 individuals (12%) met this definition for the upper bound of possible mpox cases in this age group.

In the 50 + age group of self-reported immunologically naïve participants, E8 antibodies were detected in 72 of 81 individuals (89%). Using the same case definition as for MVA-vaccinated individuals in this age group, 12 individuals (15%) met the definition for the upper bound of possible mpox cases in this age group.

### Smallpox childhood vaccination

Although we collected questionnaire data on childhood smallpox vaccination, the ability to reliably recall this vaccination is limited. Of the 779 respondents aged less than 50 years in our sample, 236 self-reported smallpox vaccination as a child, despite growing up in a time when childhood smallpox vaccination had already been discontinued in most countries. We therefore decided not to use these questionnaire data for classification, but to make the conservative assumption for our strict case definition that any serological predictions of mpox infection in study participants aged 50 years and older might be biased by antibodies induced by childhood smallpox vaccination, unless we had a report of a clinical diagnosis of mpox. Among the 70 participants with a reported mpox diagnosis, there were eight survey participants aged 50 years and older. For estimating the upper bound of potential mpox infections in participants 50 years and older we used a 100% specificity cut-off for ATI-N ab, which would rarely if at all be surpassed in people having received a smallpox vaccination more than 50 years ago. Survey participants younger than 50 years were generally assumed not to have received a childhood smallpox vaccine. A small number of participants in their late 40s may have been misclassified by this assumption, particularly because some countries in Eastern Europe stopped childhood smallpox vaccination later than countries in Western Europe, and we did not have information on the country of origin of the study participants. The total number of participants classified as suspected mpox infection in the 40–49 age group was 22, representing 13% of the participants in this age group. In the lower age groups the proportions were 14% in the 30–39-year age group and 11% in the 18-29-year age group.

Unfortunately, age and reported data on mpox diagnosis and MVA vaccination were missing for 176 participants among whom 38 (22%) met the definition for the upper bound of potential mpox cases in this group, but none was included in the strict case definition.

### Reported symptoms without mpox diagnostics

All study participants without a clinical diagnosis of mpox were asked if they had experienced mpox-related symptoms without being tested for mpox. This question was answered positively by 18 study participants, of whom 15 (83%) had detectable E8 antibodies and seven (39%) had ATI-N antibodies (upper bound definition). Of these 15, 13 (87%) had received MVA vaccination(s) and two (13%) had not. These two and a further three who had received MVA (= 5/15, 33%) were classified as suspected mpox infection on the basis of E8 and ATI-N reactivity (strict case definition).

### Serologically suspected mpox infections

We defined all cases younger than 50 years with no reported diagnosis of mpox and serological reactivity to E8 and ATI-N according to our case definition as serologically suspected mpox infections. This definition included 88 individuals.

After excluding all participants without questionnaire data (*n* = 391), participants aged 50 years and older (*n* = 120, due to uncertainty regarding the origin of anti-OPXV antibodies), and participants with ATI-N reactivity but not meeting the stricter 95% specificity requirements (*n* = 58), 549 participants could be included in univariable and multivariable regression analysis. These consisted of *n* = 41 with self-reported mpox diagnosis, *n* = 47 with suspected undiagnosed mpox infection, *n* = 294 recipients of an MVA vaccination, and *n* = 167 participants who reported neither mpox diagnosis nor MVA vaccination.

Descriptive analysis showed good comparability between the mpox reported and mpox suspected groups with respect to the mpox exposure risk variables of sexual contact with persons diagnosed with mpox, number of partners, and visit to sex venues. The respective proportions for the mpox suspected group are closer to those for the mpox reported group than for the MVA group (see Table 3).

For multivariable logistic regression analysis, we combined the mpox reported and suspected groups and compared them with the combined MVA/presumed naïve participants. HIV diagnosis and PrEP use were not included in the multivariable model. HIV diagnosis was not significant in univariable analysis, PrEP use was not included because it was strongly correlated with condomless anal intercourse, thus it facilitates condomless anal intercourse and is not an mpox infection risk factor on its’ own. From the two variables measuring the use of gay sex venues for finding sexual partners we choose the categorical variable for the multivariable model. There were two variables quantifying the potential exposure risk: the number of sex partners and the number of partners with condomless anal sex. Both variables are correlated, but they are sufficiently different because there is still a proportion of men with multiple partners who preferentially use condoms instead of PrEP. Using both variables we can show that the risk for mpox is primarily associated with condomless anal intercourse, not with the number of sexual partners. In the multivariable analysis, reported sexual contact with people diagnosed with mpox, and two or more condomless anal sex partners in the past three months remained significantly positively associated with mpox infection (see Table 3).

As a sensitivity analysis, we also performed two separate analyses comparing only those with reported mpox and those with suspected mpox with the combined MVA/presumed naïve participants. The role of sexual contact with a partner diagnosed with mpox was stronger for the mpox reported compared with the mpox suspected group, but the number of condomless anal sex partners was of similar importance for both groups (see Supplementary Table S2).

**Table 3:** Associations between demographic and behaviour variables and risk for mpox infection, Berlin mpox seroprevalence study 2023

						Univariable (*N* = 549)			Multivariable (*N* = 549)		
		Mpox reported (*N* = 41)	Mpox suspected (*N* = 47)	MVA* (*N* = 294)	Naïve (*N* = 167)	OR	95% CI	p	OR	95% CI	p
Age group	**18–29**	10	18	71	84	ref			ref		
	**30–39**	21	18	148	57	1.05	0.62–1.80	0.848	0.81	0.44–1.49	0.488
	**40–49**	10	11	75	26	1.15	0.61–2.13	0.656	0.85	0.42–1.71	0.658
	**50+**	excluded									
HIV diagnosed		5 (13%)	8 (19%)	*33 (12%)*	7 (5%)	1.81	0.89–3.49	0.085			
PrEP use	**Daily**	26 (72%)	15 (39%)	158 (61%)	28 (18%)	**1.79**	1.02–3.23	**0.048**			
	**On demand**	5 (14%)	9 (23%)	54 (21%)	18 (11%)	1.58	0.74–3.27	0.227			
Social contact** with Mpox	**No**	11 (27%)	25 (53%)	138 (47%)	129 (77%)	Ref			ref		
	**Yes**	14 (34%)	7 (15%)	128 (44%)	29 (17%)	1.37	0.80–2.32	0.242	1.04	0.57–1.86	0.902
Sexual contact**		16 (39%)	15 (32%)	28 (10%)	9 (5%)	**4.61**	2.45–8.62	**< 0.001**	**2.87**	1.39–5.84	**0.004**
Sexual partners (last three months)	**None or 1**	4 (10%)	5 (11%)	26 (9%)	31 (20%)	Ref.			ref		
	**2–4**	11 (27%)	12 (27%)	101 (35%)	73 (48%)	0.84	0.38–2.00	0.673	0.46	0.16–1.36	0.147
	**5–10**	7 (17%)	11 (24%)	107 (37%)	39 (26%)	0.78	0.34–1.92	0.571	**0.18**	0.05–0.66	**0.009**
	**> 10**	19 (46%)	17 (38%)	57 (20%)	9 (6%)	**3.45**	1.59–8.19	**0.003**	0.29	0.06–1.23	0.096
Condomless anal sex partners (last three months)	**None or 1**	8 (20%)	10 (23%)	107 (38%)	91 (62%)	Ref.			ref		
	**2–4**	11 (27%)	10 (23%)	96 (34%)	43 (29%)	1.66	0.85–3.26	0.135	**2.21**	1.02–5.04	**0.049**
	**5–10**	8 (20%)	10 (23%)	49 (17%)	11 (8%)	**3.30**	1.61–6.78	**0.001**	**5.93**	2.10–18.35	**0.001**
	**> 10**	14 (34%)	13 (30%)	33 (12%)	2 (1%)	**8.49**	4.27–17.30	**< 0.001**	**9.53**	2.72–37.54	**< 0.001**
Visiting sex venues	**Yes**	30 (73%)	29 (62%)	145 (50%)	57 (34%)	**2.60**	1.62–4.25	**< 0.001**	1.65	0.91–3.01	0.102
Number of different locations	**(1–7) mean**	2.54	1.94	1.71	1.40	1.53	1.28–1.84	**< 0.001**			
Intercept									0.16	0.06–0.36	**< 0.001**

* *n* = 58 participants from the MVA group with ATI-N antibodies below the 95% specificity threshold were excluded from this analysis; **for the question wording see Q8, Supplementary File 1

### Association of antibody responses with number of vaccinations and reported number of partners

To further analyse the association between MVA vaccination, partner number and OPXV E8 and ATI-N reactivity, we investigated whether there was any evidence of an effect of reported partner number (= mpox exposure risk) on anti-OPXV antibody prevalence among study participants reporting MVA vaccination. In both subgroups of participants, with two or only one MVA vaccination, and younger than 50 years, we found an increasing OPXV ATI-N antibody prevalence with increasing number of partners (combining the two subgroups we tested the association with partner numbers in a Chi² test, which was highly significant *p* < 0.001), suggesting that undiagnosed Mpox infections may have contributed to the OPXV antibody prevalence (see Table 4).

**Table 4:** OPXV antigen exposure and potential mpox exposure in MVA recipients as predictors of OPXV AB prevalence

OPXV antigen exposure	Age group	Number of condomless anal partners in last three months	Proportion with OPXV E8 antibodies	Proportion with ATI-*N* antibodies (41% sensitivity, 95% specificity)
Mpox reported			69/70 (99%)	34/70 (49%)
MVA 2 shots	> 50 years		47/48 (98%)	n.i.
	≤ 50 years	≥ 10 partners	35/38 (92%)	7/38 (18%)
		2–10 partners	145/156 (93%)	6/156 (4%)
		0–1	86/90 (96%)	0/90 (0%)
MVA 1 shot	> 50 years		34/36 (94%)	n.i.
	≤ 50 years	≥ 10 partners	8/12 (67%)	2/12 (17%)
		2–10 partners	28/35 (80%)	3/35 (9%)
		0–1	15/27 (56%)	1/27 (4%)
Naïve	> 50 years		72/81 (89%)	n.i.
Naïve	≤ 50 years		66/285 (23%)	19/285 (7%)

n.i. = not interpretable because ATI-N antibodies may be residual from childhood smallpox vaccination

## Discussion

To our knowledge, this is the first cross-sectional, population-based study of mpox seroprevalence in the most at-risk population of an mpox hotspot city. Other studies reporting seroprevalence data excluded individuals with clinically diagnosed mpox infection and MVA vaccination and/or included pre-selected participants [19, 21], making it difficult to extrapolate prevalence to a larger population.

OPXV E8 antibodies were detected in serum samples from individuals who denied mpox diagnosis and MVA vaccination and who were maybe too young to have received childhood smallpox vaccination. The most likely explanation for these antibodies is undiagnosed mpox infection. OPXV ATI-N antibodies in MVA recipients and participants under 50 years of age who deny a mpox diagnosis are likely to identify a minimum number of people with undiagnosed mpox infection, particularly because for MVA recipients we used an antibody detection threshold with a sensitivity of just over 40%, i.e. we probably underestimate mpox infections in MVA recipients. As ATI-N reactivity was not detected in all individuals with a reported clinical diagnosis of mpox, the true number of undiagnosed Mpox infections in our sample may be even higher.

A reliable serological distinction between infection- and vaccination-induced antibodies was not possible in individuals with childhood smallpox vaccination. We nevertheless tried to estimate the number of mpox infections in participants 50 years of age and above by requiring a high anti-ATI-N ab reactivity (above a 100% specificity threshold), which would exclude most participants with ATI-N reactivity due to smallpox childhood vaccination decades ago.

Given the epidemiological dynamics and the start of the vaccination campaign (see Fig. 1), we believe that most of the undiagnosed infections occurred by the end of July 2022 and that vaccine protection became a protective factor for MSM in Berlin only after August 2022. This assumption is supported by the results of an extensive screening study among Berlin checkpoint clients, which showed that clinically inapparent mpox infections were detected in the same time frame as clinically diagnosed infections [38]. Authors from other countries have also reported MVA recipients who were serologically positive for OPXV ab at baseline screening before MVA vaccination [19, 20, 39]. This does not exclude the possibility that suspected undiagnosed infections were acquired after September 2022 and/or after MVA vaccination. A shortened course of infection and attenuation of symptoms in vaccinated individuals could also be a reason for failure to clinically recognise mpox infections. The comparatively high proportion of suspected undiagnosed infections in our sample compared with other publications could also be related to the longer period between the peak of the outbreak and sample collection. The higher ATI-N reactivity among MVA recipients with fewer than 10 condomless anal sex partners in the last 3 months and one vaccine dose compared with two vaccine doses might indicate a higher proportion of post-vaccination breakthrough infections in this subgroup. Factors significantly associated with mpox infection in a multivariable regression model were having two or more partners with whom condomless anal intercourse was practised in the past three months and reporting sexual contact with people known to have mpox. The risk of exposure to mpox in gay sex venues reported by people with suspected undiagnosed infection ranged between the risk reported by people with diagnosed infection and people vaccinated with MVA. Thus, in addition to the absence of symptoms or mild symptoms, lower risk perception may be a factor contributing to the failure to clinically diagnose infections.

### Limitations

There was a subgroup of study participants with missing data on age, history of mpox infection and vaccination which did not allow us to further classify these cases based on their ab patterns. The proportion of anti-E8 ab positivity was 68% among the 176 participants with missing data (16% of all participants). Anti-E8- ab positivity was thus almost identical among participants with missing data and the whole study sample (656/943 = 70%), suggesting that data were missing at random.

As the diagnosis of mpox and the history of MVA vaccination were collected by a self-administered questionnaire, recall bias cannot be excluded. However, we believe that these data are highly reliable because we asked about infections and vaccinations that had occurred in the previous 9–11 months and would generally be well remembered.

Due to the performance of our multiplex assay for ATI-N detection, we decided to use a compromise between specificity and sensitivity of ATI-N ab detection for our case definition of suspected undiagnosed infections. Thus, we are likely to underestimate the true number of infections in the subgroup of participants vaccinated with MVA because we used a threshold that has a sensitivity of only 41%, and because not all true infections would be ATI-N ab positive. Using this stricter definition, 41% (61/149) of the estimated mpox infections would have been clinically diagnosed (see Table 2). We also tried to estimate an upper range for undiagnosed mpox infections by using a lower threshold for anti ATI-N ab. Using this upper range estimate only 25% (70/276) of the estimated mpox infections would have been clinically diagnosed.

It is likely that our strict case definition underestimated the number of undiagnosed mpox infections in the age groups 50 years and older because we assumed that all OPXV ab in these age groups were due to childhood smallpox vaccination, unless participants reported clinical mpox diagnoses. As eight of the 70 reported diagnoses were in people aged 50 and over, it is likely that there were some undiagnosed infections in this age group. On the other hand, some of the people with suspected mpox infections in the age group 40–49 may have been misclassified because they may have received the smallpox vaccine as children. It might have been preferable to document vaccination scars instead of asking for smallpox childhood vaccination. However, based on the very similar proportions with serologically suspected mpox infection in other age groups, we believe that the number of ppts aged 40–49 years who did receive smallpox vaccination was small and probably did not exceed a handful of individuals. Last but not least, we are unable to exclude the possibility that individual cases of anti-E8 and anti-ATI-N seropositivity could be due to ab against cross-reacting orthopoxviruses other than MPXV.

## Conclusion

Based on our serological test results from the Berlin MSM sample, we estimate that only between 25 and 41% of individuals with mpox infection were clinically diagnosed. More research will be necessary to determine the timing of mpox infections in people vaccinated with MVA, e.g. by analysing anti-ATI-N reactivity in serum samples taken before and after MVA vaccination. Only a small proportion of those with serological suspicion but no clinically confirmed diagnosis of mpox reported experiencing symptoms consistent with mpox infection. While we cannot exclude the possibility that a larger proportion of these individuals had mild clinical symptoms that did not raise suspicion and trigger further diagnostic efforts, it is clear that in real life a significant proportion of mpox infections will remain clinically undiagnosed. With such a high proportion of undiagnosed infections, it is highly likely that undiagnosed infections will play an important role in the spread of the virus in outbreak situations. Quarantine or other drastic contact restrictions for people diagnosed with mpox, or who have had social or sexual contact with people diagnosed with mpox, may therefore have a very limited impact on outbreak containment. Rapid and unrestricted access to the MVA vaccine is the best option for controlling the spread of mpox. In the absence of vaccine availability, condom use for anal/vaginal intercourse is likely to be protective against sexual transmission, based on our data, which identify the number of condomless anal sex partners, rather than the number of sexual partners, as the main risk factor for mpox infection.

Our findings raise serious doubts that mpox can ever be eradicated with currently available tools once the virus has established itself in a susceptible population in a country. Following the global mpox outbreak among MSM in 2022, it is therefore unlikely that mpox will disappear from this population in the foreseeable future.

## Electronic supplementary material

Below is the link to the electronic supplementary material.

Supplementary Material 1

Supplementary Material 2

Supplementary Material 3

## Data Availability

The data that support the findings of this study are available from Robert Koch Institute, but restrictions apply to the availability of these data, which are not publicly available. The data are, however, available from the authors upon reasonable request and with the permission of Robert Koch Institute (RKI).
