# Correction: A seroprevalence study indicates a high proportion of clinically undiagnosed MPXV infections in men who have sex with men in Berlin, Germany

**DOI:** 10.1186/s12879-024-10101-z

**Published:** 2024-10-28

**Authors:** Ulrich Marcus, Janine Michel, Nikolay Lunchenkov, Denis Beslic, Fridolin Treindl, Rebecca Surtees, Christoph Weber, Axel Baumgarten, Andreas Nitsche, Daniel Stern

**Affiliations:** 1 Department of Infectious Disease Epidemiology, Robert Koch Institute, Berlin, Germany; 2 Centre for Biological Threats and Special PathogensGerman Consultant Laboratory for Poxviruses Highly Pathogenic Viruses (ZBS 1) WHO Collaboration Center for Emerging Threats and Special Pathogens, Berlin, Germany; 3 Technical University of Munich, TUM School of Social Sciences and Technology, Munich, Germany; 4 Centre for Biological Threats and Special Pathogens, Biological Toxins (ZBS 3), Robert Koch Institute, Berlin, Germany; 5 Centre for Artificial Intelligence in Public Health Research, ZKI-PH 3, Wildau, Germany; 6 Checkpoint BLN, Berlin, Germany; 7 MVZ zibp, Berlin, Germany

**Correction: BMC Infectious Diseases (2024) 24:1153**

10.1186/s12879-024-10066-z

Following publication of the original article [1], we were notified that a second affiliation was missed for Nikolay Lunchenkov: Technical University of Munich, TUM School of Social Sciences and Technology, Munich, Germany.

The original article has been corrected.
