# Supplementary material for: A seroprevalence study indicates a high proportion of clinically undiagnosed MPXV infections in men who have sex with men in Berlin, Germany
Source: BMC Infect Dis. 2024 Oct 14;24:1153. doi: 10.1186/s12879-024-10066-z (PMC11472563; doi:10.1186/s12879-024-10066-z)
Supplement: Supplementary file 1 — Supplementary Material 1 [file 12879_2024_10066_MOESM1_ESM.docx]

**Supplementary File 1**

| **Online Questionnaire, Mpox seroprevalence study, Berlin** | |
| --- | --- |
| Q1 How old are you? Please select age group. | |
| 18-29 | |
| 30-39 | |
| 40-49 | |
| 50-59 | |
| 60+ | |
|  | |
| Q2 What gender were you assigned at birth? | |
| Masculine | |
| Feminine | |
| I don't want to say | |
|  | |
| Q3 What is your current gender identity? | |
| Male | |
| Female | |
| Trans* male | |
| Trans* female | |
| Non-binary | |
| Other | |
|  | |
| Q4 The following 3 statements are correct | |
| So far in Germany, almost exclusively men who have sex with men have been affected by "Mpox (monkeypox)". | |
| I already knew that | |
| I wasn't sure about that | |
| I did not know that | |
| I don't understand the statement | |
| I don't think that's true | |
| Mpox (monkey pox) is mainly transmitted during sex and close physical contact. | |
| I already knew that | |
| I wasn't sure about that | |
| I did not know that | |
| I don't understand the statement | |
| I don't think that's true | |
| There is a vaccine against "Mpox (monkeypox)" that significantly reduces the risk of contracting the disease. | |
| I already knew that | |
| I wasn't sure about that | |
| I did not know that | |
| I don't understand the statement | |
| I don't think that's true | |
|  | |
| Q5 Have you been diagnosed with Mpox (monkeypox) before? | |
| Yes | |
| No | |
|  | |
| Q6 Date of Diagnosis (MM/YYYY) | |
|  | |
| Q7 Have you had symptoms that indicated monkeypox infection without a laboratory test and medical diagnosis? | |
| Yes | |
| No | |
| I don't want to answer | |
|  | |
| Q8 Do you know someone who has been diagnosed with "Mpox (monkeypox)"? | |
| No, I don't know anyone | |
| Yes, one or more people I have had sex with | |
| Yes, one or more people I know but have not had sex with | |
| I don't want to say that | |
|  | |
| Q9 How worried are you about catching Mpox (monkeypox)? | |
| Not worried at all | |
| Just a little concerned | |
| Pretty worried | |
| Very concerned | |
| I don't know it | |
|  | |
| Q10 Have you received a vaccination against Mpox (monkeypox) since June 2022? | |
| Yes | |
| No | |
| I don't know | |
|  | |
| Q11 Number of Mpox (monkeypox) vaccinations | |
| 1 | |
| 2 | |
| I don't know | |
|  | |
| Q12 Date of last vaccination against Mpox (monkeypox) (MM/YYYY) | |
|  | |
| Q13 If you were offered a vaccine for 'Mpox (monkey pox)', would you be vaccinated? | |
| I would not get vaccinated | |
| Probably not | |
| I am not sure | |
| Probably yes | |
| I would get vaccinated | |
|  | |
| Q14 Why haven't you been vaccinated yet? (check all that apply) | |
| It was too complicated to arrange a vaccination appointment | |
| I don't think I need a vaccination because I don't think I'm at risk | |
| I find vaccination unnecessary for me because the number of new infections has already fallen sharply | |
| I find vaccination unnecessary for me because I have already gone through an infection | |
| I'm afraid of side effects | |
| I would get vaccinated later in case monkeypox spread again | |
|  | |
| Q15 Did you get a smallpox vaccine when you were a child? | |
| Yes | |
| No | |
| I don't know | |
|  | |
| Q16 Number of smallpox vaccinations | |
| 1 | |
| 2 | |
| I don't know | |
|  | |
| Q17 To what extent do you agree with the following statement: "Vaccinations protect us from many diseases" ? | |
| I don't agree at all | |
| I tend not to agree | |
| I tend to agree | |
| I totally agree | |
| I don't know it | |
|  | |
| Q18 Have you changed your sexual behavior/your choice of partner since you found out about  "Mpox (monkey pox)"? | |
| Yes | |
| At first yes, but not anymore | |
| No | |
|  | |
| Q19A If so, where did you limit yourself? [Please select] | |
| The number of people I have sex with | |
| Visiting places/events where spontaneous sex is possible | |
| Anal intercourse | |
| Oral sex | |
| Vaginal intercourse | |
|  | |
| greatly reduced | |
| somewhat reduced | |
| unchanged | |
| does not apply | |
|  | |
| Q19B What have you done more often? [Please check] | |
| Using condoms during anal intercourse | |
| Raised the subject of monkeypox | |
| Contact information exchanged to notify each other | |
|  | |
| more often | |
| unchanged | |
| does not apply | |
|  | |
| Q19C Did you change anything else? | |
| Yes | |
| No | |
|  | |
| Q20 In this survey, by "sex" we mean physical contact aimed at the orgasm of one or both partners.  With which partners did you have sex in the last 3 months (multiple answers possible)? | |
| With men | |
| With women | |
| With non-binary/trans people | |
|  | |
| Q21a/b/c With how many different male/ female/ non-binary partners have you had sex in the last 3 months? | |
| None | |
| 1 | |
| 2 | |
| 3 | |
| 4 | |
| 5-7 | |
| 8-10 | |
| 11-20 | |
| 21-30 | |
| 31-40 | |
| 41-50 | |
| More than 50 | |
|  | |
| Q22a/b/c With how many different male/ female/ non-binary partners have you had anal sex without a condom  in the last 3 months? | |
| None | |
| 1 | |
| 2 | |
| 3 | |
| 4 | |
| 5-7 | |
| 8-10 | |
| 11-20 | |
| 21-30 | |
| 31-40 | |
| 41-50 | |
| More than 50 | |
|  | |
| Q23 In what context did most of the sexual encounters of the last 3 months take place  (multiple answers possible)? | |
| Private with one/two partners (no group sex) | |
| Private sex party | |
| Public sex party | |
| In clubs or darkrooms | |
| In saunas | |
| In porn cinemas | |
| At cruising locations | |
| In other places | |
|  | |
| Q24 Have you ever received an HIV test result? | |
| Yes | |
| No | |
| I do not know | |
|  | |
| Q25 Have you ever been diagnosed with HIV? | |
| Yes | |
| No | |
| I don't want to say | |
|  | |
| Q26 Have you taken PrEP in the last 3 months? | |
| No | |
| Yes, daily, and I take it up to date | |
| Yes, daily, but I don't take them at the moment | |
| Yes, if necessary, but not every day | |
| I do not know anymore | |
