# Supplementary material for: A seroprevalence study indicates a high proportion of clinically undiagnosed MPXV infections in men who have sex with men in Berlin, Germany
Source: BMC Infect Dis. 2024 Oct 14;24:1153. doi: 10.1186/s12879-024-10066-z (PMC11472563; doi:10.1186/s12879-024-10066-z)
Supplement: Supplementary file 2 — Supplementary Material 2 [file 12879_2024_10066_MOESM2_ESM.docx]

Supplementary Table S1: Summary of required/expected OPXV antibody patterns

|  | **Serologically suspected mpox** (case definition) | **Upper bound** for potential mpox infections | **No mpox** (expected antibody patterns) |
| --- | --- | --- | --- |
| MVA, age <50 | anti-E8 +, anti-ATI-N + (>=95%spec./41%sens. threshold) | anti-E8 +, anti-ATI-N + (>=77% spec./78% sens. threshold) | anti-E8 -, anti-ATI-N - |
| >=50 (assumed smallpox childhood vaccination) | not included | Anti-E8 +, anti-ATI-N + (>=100% specificity threshold) | anti-E8 +, anti-ATI-N + <100% specificity possible |
| No MVA, age < 50 | anti-E8 + | = serologically suspected | anti-E8 -, anti-ATI-N - |
| >=50 (assumed smallpox childhood vaccination) | not included | Anti-E8 +, anti-ATI-N + (>=100% specificity threshold) | anti-E8 +, anti-ATI-N + <100% specificity possible |
