# Supplementary material for: A seroprevalence study indicates a high proportion of clinically undiagnosed MPXV infections in men who have sex with men in Berlin, Germany
Source: BMC Infect Dis. 2024 Oct 14;24:1153. doi: 10.1186/s12879-024-10066-z (PMC11472563; doi:10.1186/s12879-024-10066-z)
Supplement: Supplementary file 3 — Supplementary Material 3 [file 12879_2024_10066_MOESM3_ESM.docx]

Supplementary Table S2

Separate multivariable regression analyses for reported mpox cases and serologically suspected mpox cases

|  |  | Multivariable (N=502, mpox diagnosed) | | | Multivariable (N=508, mpox suspected) | | |
| --- | --- | --- | --- | --- | --- | --- | --- |
|  |  | OR | 95% CI | p | OR | 95% CI | p |
| Age group | **18-29** | ref |  |  | ref |  |  |
|  | **30-39** | 1.07 | 0.45 - 2.65 | 0.885 | 0.65 | 0.30 - 1.43 | 0.284 |
|  | **40-49** | 1.03 | 0.37 - 2.82 | 0.955 | 0.73 | 0.29 - 1.77 | 0.493 |
| Social contact with MPXV | **No**  **Yes** | Ref  1.50 | 0.63 - 3.62 | 0.363 | ref  0.78 | 0.36 - 1.62 | 0.505 |
| Sexual contact |  | **7.45** | 2.95 – 19.44 | **<0.001** | 1.21 | 0.39 – 3.33 | 0.720 |
| Sexual partners (last three months) | **None or 1**  **2-4**  **5-10**  **>10** | Ref.  0.23  0.06  0.13 | 0.05 - 1.07  0.01 - 0.37  0.01 - 1.03 | 0.056  **0.004**  0.060 | ref  0.76  0.40  0.46 | 0.20 - 3.74  0.07 - 2.35  0.06 - 3.54 | 0.710  0.282  0.451 |
| Condomless anal sex partners (last three months) | **None or 1**  **2-4**  **5-10**  **>10** | Ref.  3.01  **7.14**  **8.66** | 0.94 – 11.62  1.47 - 43.83  1.52 – 63.94 | 0.079  **0.021**  **0.023** | ref  1.79  **5.74**  **12.26** | 0.65 – 5.17  1.55 – 24.77  2.39 – 80.38 | 0.265  **0.012**  **0.005** |
| Sex venues | **Yes** | **2.71** | 1.15 - 6.72 | **0.026** | 1.20 | 0.56 - 2.60 | 0.634 |
| Intercept |  | **0.06** | 0.01 – 0.18 | **<0.001** | **0.08** | 0.02 – 0.26 | **<0.001** |
